# Does long-term care insurance affect the length of stay in hospitals for the elderly in Korea?: a difference-in-difference method

**DOI:** 10.1186/s12913-014-0630-1

**Published:** 2014-12-21

**Authors:** Kyung-Rae Hyun, Sungwook Kang, Sunmi Lee

**Affiliations:** Health Insurance Policy Research Institute, National Health Insurance Service, Mapo-gu, Seoul South Korea; School of Public Health, Daegu Haany University, Gyeongsan-si, Gyeongsangbuk-Do South Korea

**Keywords:** Long-term care insurance, Length of stay, Difference-in-difference

## Abstract

**Background:**

This study examines the effects of long-term care insurance (LTCI) on the length of stay (LoS) of senior citizens under the national health insurance of Korea.

**Methods:**

The subjects include 3,903,448 people aged 65 and over as of July 1, 2008 when the LTCI was introduced in Korea. This study uses their panel data which traced the records of medical services and LTCI services for the same people from 2007 to 2010, and applies a difference-in-difference approach on LTCI users from levels 1, 2, and 3 who are the treatment group and non-LTCI users who are the control group.

**Results:**

We found that the LoS of LTCI users is 1.27 days greater than that of non-LTCI users, but the LoS of level 1 and level 2 beneficiaries decreases by 8.35 and 2.84 days, respectively, whereas the LTCI does not reduce the LoS of level 3 beneficiaries.

**Conclusions:**

The reason why there is an effect on the LoS of level 1 and 2 beneficiaries is that these groups could choose to utilize institutional care services provided by the LTCI, and out-of-pocket costs of institutions are lower than that of hospitals. However, the reason why there is no effect on the LoS of level 3 beneficiaries is that they are not permitted to use the institutional care services in the Korean LTCI policy. Therefore, we recommend a modification in the LTCI system that facilitates the use of long-term care institutional services by level 3 beneficiaries without conflicting Korea’s LTCI principle to promote home-based care services instead of the institutional care services.

## Background

The national health insurance (NHI) program of Korea provides coverage for the entire population under a compulsory health insurance scheme. Insurance benefits are provided to the insured and their dependents to cover the prevention and treatment of sickness. Health care expenditure as a percentage of GDP in Korea was approximately 7.1% in 2010, while the average annual increase rate in health care expenditure was 9.1% from 2000 to 2009, the greatest increase rate among 33 OECD countries [1]. The Korean government paid special attention to the aging population, which it believed may cause a sharp increase in health care expenditure. The proportion of people aged 65 and over was 11.0% in 2010 [2], and this group accounted for 32.4% of all health care expenditure in Korea [3].

The number of chronic-care beds for the elderly population was insufficient in Korea until the early 2000s. The Korean government did not make preparations for augmenting the number of chronic-care beds even though the demand for chronic-care beds was expected to increase because of the aging population. Accordingly, as inappropriate hospitalizations in acute-care beds for seniors led to longer stays, this environment strained the Korean NHI system with higher costs for hospital treatment compared to nursing care services in residential areas. To overcome this situation, the Korean government introduced a new social insurance scheme for long-term care in July 2008, which was based on a pilot study conducted in several regions throughout the country. Hence, there are two mandatory insurances in Korea; one is the NHI program, which provides medical care services; the other is the long-term care insurance (LTCI) program, which provides social services for the elderly with a few functional limitations. One of the aims of the LTCI program is to reduce the utilization of inappropriate health care services by elderly patients within the NHI program. Therefore, it is important to analyze the effect of the LTCI program on hospitalizations relating to the NHI program for the elderly population in Korea.

Many studies have evaluated the quantitative and qualitative aspects of the Korean LTCI program, such as changes in the scope of benefits [4-6], the economic burden and quality of life for beneficiaries and family caregivers [7-11], and job satisfaction rates for long-term care workers [12]. However, few studies have examined the effects of the Korean LTCI program on the utilization of health care services covered by the Korean NHI program for seniors. There are a few studies suggesting the existence of a relationship between long-term care and acute care based on results of other countries [13-16]. Forder [15] demonstrates that since home-based or institutional social services for the frail and elderly population could reduce the rate of admissions to hospitals, the LTCI program could facilitate more timely discharge from hospitals. Tomita *et al*. [17] suggests that the use of home-based and community-based services decrease the probability of hospitalization and institutionalization of the aged population by diminishing the functional or health-related risks.

Because six years have passed since the introduction of the LTCI program in Korea, it is time to evaluate the effects of the LTCI program for the elderly under the NHI program. Therefore, the purpose of this study is to examine the effects of the LTCI program on the length of stay (LoS) in all clinics and hospitals for those aged 65 years and over who are under the NHI program in Korea. This paper proceeds in the following manner: First of all, we describe the methodology and data in this study. Next, we describe the empirical results. Finally, we discuss the implications of the empirical results in the discussion section.

## Methods

### Study design

This study uses the panel data from 2007 to 2010. Our panel data traced the records of medical services and LTCI services for the same people over four years and applies the difference-in-difference (DID) method to estimate the effects of the LTCI program on the LoS for those aged 65 years and over before and after the introduction of the LTCI program [18,19]. Many studies have used the DID approach to measure the effects of policy changes or an introduction of a new policy [20-22]. The DID method is a standard policy evaluation tool that examines the effects of a policy intervention on a treatment group in comparison to a control group once a particular policy is initiated. The treatment group in this study consists of people aged 65 years or older who use the services offered by the LTCI program in Korea, while the control group consists of those aged 65 years or older who do not use the services provided by the LTCI program.

When conducting a policy analysis by using panel data, the DID method assumes that unobserved effects are the same for both the treatment group and the control group. Thus, the change in health care use in the treatment group before and after the introduction of the LTCI program, minus the corresponding change in the control group, provides an estimate of the impact of the LTCI program on health care use. The identifying assumption of the DID estimator is that time-varying factors affect both groups equally. This study examines four time periods from each individual, and the unobserved effects panel data model is as follows:

$$ {\mathrm{H}}_{\mathrm{i}\mathrm{t}} = {\updelta}_1+{\updelta}_2\mathrm{D}2+{\updelta}_3\mathrm{D}3+{\updelta}_4\mathrm{D}4+{\upbeta}_1{\mathrm{LTCI}}_{\mathrm{i}\mathrm{t}}+{\upbeta}_2{\mathrm{Z}}_{\mathrm{i}\mathrm{t}}+{\mathrm{a}}_{\mathrm{i}}+{\mathrm{u}}_{\mathrm{i}\mathrm{t}} $$

where H_it_ is a patient’s health care use, such as LoS, for person i at time t. D2 is the year dummy for 2008 and D3 for 2009 and D4 for 2010. The binary variable LTCI_it_ is equal to one if person i at time t received benefits from LTCI, while Z_it_ represents a variety of socio-economic characteristics (e.g., age, sex, NHI premium, residence, health status, and medical services utilization). The unobserved effect is represented by a_i_. The unobserved effect a_i_ represents fixed factors that affect the functional condition in person i. Because LTCI eligibility is not determined randomly – LTCI users often have functional limitations – it is likely that LTCI_it_ and a_i_ are positively correlated. Thus, we difference the equation to eliminate a_i_. This yields

$$ \Delta {\mathrm{H}}_{\mathrm{it}} = {\updelta}_2\Delta \mathrm{D}2+{\updelta}_3\Delta \mathrm{D}3+{\updelta}_4\Delta \mathrm{D}4+{\upbeta}_1\Delta {\mathrm{LTCI}}_{\mathrm{it}}+{\upbeta}_2\Delta {\mathrm{Z}}_{\mathrm{it}}+\Delta {\mathrm{u}}_{\mathrm{it}} $$

for t = 2, 3, and 4. Because age and sex in ΔZ_it_ are time-constant variables, they are eliminated.

The above equation contains the first differences in the year dummies, D2 and D3 and D4 but does not contain an intercept. This is inconvenient for certain purposes, including the computation of R-squared. Because we are not interested in time intercepts in the above equation, it is generally better to estimate the first-differenced equation with an intercept and a double time period dummy (ΔD3, ΔD4) for the four periods. In other words, the final structural equation becomes:

$$ \Delta {\mathrm{H}}_{\mathrm{it}} = {\upalpha}_1+{\updelta}_3\Delta \mathrm{D}3+{\updelta}_4\Delta \mathrm{D}4+{\upbeta}_1\Delta {\mathrm{LTCI}}_{\mathrm{it}}+{\upbeta}_2\Delta {\mathrm{Z}}_{\mathrm{it}}+\Delta {\mathrm{u}}_{\mathrm{it}} $$

The regression of the above equation is conducted to determine the effects of the LTCI program on the LoS for LTCI users (treatment group) compared to non-LTCI users (control group). The estimate of β_1_ measures a pre-post change in the LoS for the treatment group after the introduction of the LTCI program. Also, a more detailed policy analysis is performed by separating the treatment group according to the respectively approved level in the LTCI program.

### Data

The dataset used in this study is the NHI and LTCI dataset developed by the Korean National Health Insurance Service (NHIS). The dataset include information of subscriber’s qualification and medical usage which NHIS constructed based on the ‘Article 15′ of the ‘Personal Information Protection Law’. The authors received permission to use data after deliberation of the Research Project Review Committee (RPRC) in accordance with the Research Project Management Guidelines of NHIS.

The NHI dataset is taken from 2007 to 2010, and the LTCI dataset is taken from July 2008 to December 2010. The NHI dataset used in this study contains socio-economic information like sex, age, monthly premium, classification of NHI, residence, health status, and medical services utilization. More specifically, we utilize the number of chronic diseases as proxy variable for health status and the number of physician visits as proxy variable for medical services utilization. The LTCI dataset used in this study contains information regarding the approved levels, such as level 1, level 2, and level 3, and the type of LTCI service.

The DID method can be used when the data is generated from a natural experiment where the economic environment – sometimes summarized by an explanatory variable – goes through an exogenous change, perhaps inadvertently, due to a change in policy. A natural experiment occurs when some exogenous event, e.g., the introduction of LTCI, changes the environment in which individuals, families, firms, or cities operate. A natural experiment always has a control group (non-LTCI users), which is not affected by the policy change, and a treatment group (LTCI users), which is thought to be affected by the policy change. Hence, we eliminated some data that is not produced in a natural experiment.

The subjects of this study are 4,507,821 people aged 65 and over as of July 1, 2008 when the LTCI program was introduced in Korea. We excluded 357,684 people who died from July 1, 2008 to December 31, 2010 and 144,234 recipients subsidized by the Medical Aids program. Because these individuals tend to overuse health care services, they are not suitable for inclusion in the control group. Furthermore, we eliminated 102,455 people (switchers) whose approved level was changed in the LTCI program within one year and people (non-users) who did not use LTCI services even though they received approval for their level by the local needs-assessment-committees in the LTCI programs. Because it is difficult to analyze the effect of government policy during the post-LTCI period, switchers and non-users are not suitable for inclusion in the treatment group. Based on the above exclusion criteria, the number of subjects in this study is 3,903,448 individuals. To make policy analysis with the panel data, we traced the records of their medical services and LTCI services.

### Ethics statement

This research was directly performed by Health Insurance Policy Research Institute of NHIS as the investigative research in connection with the health insurance legislated in the ‘National Health Insurance Act Article 14’. According to the ‘Bioethics and Safety Act Enforcement Regulations Article 2’, researches that nation carried out directly or commissioned to review and evaluate public welfare and service programs are exempt from ethical approval of a regional Institutional Review Board (IRB). Therefore, neither ethical approval of an IRB nor written informed consent was required for this study according to the relevant legislation because this study was directly performed by Korean public single insurer, NHIS, to evaluate Korean NHI and LTCI.

## Results

Table 1 shows the characteristics of the subjects in 2007. The classification of NHI, monthly premiums for NHI, and the subject’s residence are taken from the last day of 2007. We deflated the nominal monthly premium by using the consumer price index (CPI) of Statistics Korea (2010 = 100). The number of chronic diseases indicates primary diseases recorded in the NHI dataset which include hypertensive diseases, diabetes mellitus, heart diseases, cerebrovascular diseases, neoplasms, liver diseases, and chronic kidney diseases. The level of LTCI in the treatment group refers to the approved level of the LTCI beneficiary who remained at the same level from 2008 to 2010.

**Table 1:** **Characteristics of subjects in 2007**

	**Treatment group**				**Control group**	**Total**
	**Level 1**	**Level 2**	**Level 3**	**Subtotal**		
**Sex**						
Male	1,445 (23.5%)	1,165 (21.4%)	3,288 (24.2%)	5,993 (23.3%)	1,610,861 (41.5%)	1,616,854 (41.4%)
Female	4,700 (76.5%)	4,283 (78.6%)	10,319 (75.8%)	19,699 (76.7%)	2,266,895 (58.5%)	2,286,594 (58.6%)
**Age **(years, ±S.D.^*^)	76.96 (±7.42)	78.27 (±7.30)	77.13 (±7.02)	77.34 (±7.20)	71.31 (±5.84)	71.35 (±5.87)
**NHI classification**						
Employee	4,215 (68.7%)	3,762 (69.1%)	9,356 (68.8%)	17,658 (68.8%)	2,565,730 (66.3%)	2,583,388 (66.3%)
Self- employed	1,925 (31.4 %)	1,682 (30.9%)	4,250 (31.2%)	8,024 (31.2%)	1,303,302 (33.7%)	1,311,326 (33.7%)
**NHI premium** (ten thousand KRW, ±S.D.^*^)	9.64 (±8.56)	9.10 (±7.79)	9.11 (±8.52)	9.23 (±8.36)	8.47 (±7.65)	8.48 (±7.66)
**Residence**						
Large city	2,801 (45.6 %)	2,495 (45.8%)	6,008 (44.2%)	11,538 (44.9%)	1,740,988 (45.0%)	1,752,526 (45.0%)
Small city	2,768 (45.1%)	2,391 (43.9%)	5,944 (43.7%)	11,328 (44.1%)	1,683,043 (43.5%)	1,694,371 (43.5%)
Rural	571 (9.3%)	558 (10.3%)	1,654 (12.2%)	2,816 (11.0%)	445,001 (11.5%)	447,817 (11.5 %)
**Number of physician visits** (±S.D.^*^)	24.79 (±34.81)	26.92 (±37.62)	37.97 (±44.20)	32.37 (±41.16)	34.76 (±34.80)	34.74 (±34.85)
**Number of chronic disease** (±S.D.^*^)	2.26 (±1.56)	2.23 (±1.62)	2.31 (±1.57)	2.28 (±1.58)	1.49 (±1.34)	1.50 (±1.34)
**Total**	6,145 (100.0%)	5,448 (100.0%)	13,607 (100.0%)	25,692 (100.0%)	3,877,756 (100.0%)	3,903,448 (100.0%)

^*^S.D.: standard deviation.

*Notes*: Level 1, 2, 3 beneficiaries in 2007 was recalculated with people having the same level during 2008 ~ 2010.

58.6% of all subjects, 76.7% of the LTCI users within the treatment group, and 58.5% of non-LTCI users in the control group are female. The average age of the LTCI and non-LTCI users is 77.3 and 71.3 respectively. Among all subjects, 66.3% are classified as employee insured and 33.7% have self-employed health insurance. The monthly premium for NHI is 92 thousand Korean won (KRW) for LTCI users and 85 thousand Korean won (KRW) for non-LTCI users. With respect to residence, 45% live in large cities, 43.5% live in small cities, and 11.5% live in rural areas. The average number of physician visits is 32.4 for LTCI users and 34.8 for non-LTCI users. The average number of chronic diseases is 2.3 for LTCI users and 1.5 for non-LTCI users.

**Figure 1:**
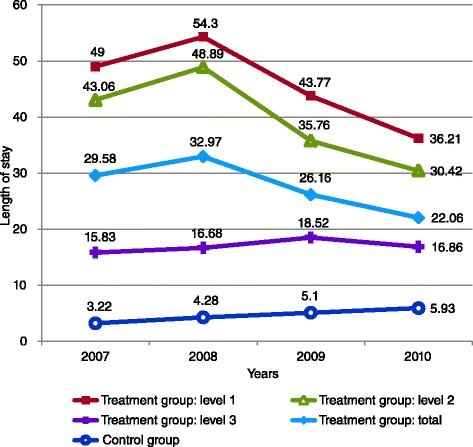
**Length of stay of treatment group and control group.**

Figure 1 shows the LoS of LTCI users and non-LTCI users. The LoS of LTCI users (treatment group) is longer than that of non-LTCI users (control group). Within the treatment group, the LoS is the longest among level 1 beneficiaries. The LoS of the control group increased over 4 years from 3.22 days in 2007 to 5.93 days in 2010, while the LoS of the treatment group increased from 29.58 days in 2007 to 32.97 days in 2008 and then decreased to 22.06 days in 2010. The fluctuations in the LoS of the total LTCI users corresponds to the fluctuations in the LoS of level 1 and level 2 beneficiaries over time. For level 3 beneficiaries, the LoS increased to 18.52 days in 2009 and then decreased to 16.86 days in 2010.

Table 2 shows the effects of the LTCI program on the LoS of LTCI users compared to non-LTCI users. Model (1) shows the effects of the LTCI program on all LTCI users, while models (2), (3), and (4) show the effects of the LTCI program on LTCI users from each level compared to non-LTCI users. The results suggest that the introduction of the LTCI program is accompanied by an increase of 1.27 days in the LoS of LTCI users when controlling for the classification of NHI, contributions of NHI, residence, the number of physician visits, and the number of chronic diseases. With respect to the levels of LTCI, the LTCI program causes the LoS of level 1 beneficiaries to decrease by 8.35 days and causes the LoS of level 2 beneficiaries to decrease by 2.84 days. For level 3 beneficiaries, the LTCI program may increase the LoS but the parameter is not statistically significant at the 5% level against a two-sided alternative. In model (1), the first-differenced year dummies representing 2009–2008 and 2010–2009 present negative signs, and the absolute values increase from 0.03 to 0.20, which means that the LoS for the elderly shows a decreasing trend over time for both the treatment and the control groups. On the whole, the LoS is positively associated with NHI premium, large city, and the number of chronic diseases. Conversely, a negative relationship between the LoS and the number of physician visits is apparent.

**Table 2:** **Effects of the LTCI program on the LoS** (**Difference**-**in**-**difference estimation**)

	**Model 1**	**Model 2**	**Model 3**	**Model 4**
	**LTCI users**	**Level 1**	**Level 2**	**Level 3**
**Intercept**	0.960*** (0.011)	0.989*** (0.010)	0.981*** (0.010)	0.965*** (0.010)
**Treatment** (ref: Control)	1.271*** (0.059)	−8.349*** (0.130)	−2.843*** (0.114)	0.108 (0.067)
**2009**-**2008** (ref: 2008–2007)	−0.028* (0.016)	−0.018 (0.015)	0.002 (0.015)	0.062*** (0.015)
**2010**-**2009** (ref: 2009–2008)	−0.203*** (0.027)	−0.178*** (0.025)	−0.138*** (0.025)	−0.034 (0.026)
**Employee** (ref: Self-employed)	0.045* (0.024)	0.023 (0.023)	0.034 (0.023)	0.026 (0.023)
**NHI premium** (ten thousand KRW)	0.013*** (0.001)	0.012*** (0.001)	0.013*** (0.001)	0.013*** (0.001)
**Large city** (ref: Non-large city)	0.104*** (0.031)	0.108*** (0.029)	0.102*** (0.029)	0.104*** (0.029)
**Number of physician visits**	−0.038*** (0.000)	−0.032*** (0.000)	−0.033*** (0.000)	−0.034*** (0.000)
**Number of chronic diseases**	2.268*** (0.006)	2.086*** (0.005)	2.090*** (0.005)	2.120*** (0.005)
**Adj R**-**Square**	0.014	0.013	0.013	0.013
**Subjects size**	n = 11,626,010	n = 11,438,550	n = 11,447,639	n = 11,546,567

*significant at the 10% level, **significant at the 5% level, ***significant at the 1% level.

*Notes*: (Model 1) treatment group = LTCI users, control group = non-LTCI users; (Model 2) treatment group = LTCI users in level 1, control group = non-LTCI users; (Model 3) treatment group = LTCI users in level 2, control group = non-LTCI users; (Model 4) treatment group = LTCI users in level 3, control group = non-LTCI users.

## Discussion

This study reveals that the introduction of a LTCI program in Korea is not effective in reducing the LoS of LTCI users as was expected to decrease because the LoS of LTCI users is 1.27 days greater than the LoS of non-LTCI users. There are two main reasons for this result. Firstly, the admission days of level 3 beneficiaries are not expected to be reduced because they are not permitted to use the institutional care services provided by the Korean LTCI program except in the case that they have no family support or live in an inadequate housing condition. With respect to the medical conditions of LTCI beneficiaries, the primary disease in each level is dementia. Level 1 beneficiaries account for 22%, level 2 accounts for 26.9%, and level 3 accounts for 22.6% [23]. Those beneficiaries whose main disease consist of a stroke accounted for 33.3% in level 1, 25.4% in level 2, and 23.2% in level 3 [23]. Concerning the need for long-term care, most notably nursing care, there is no difference between the three levels [24]. Under the circumstance in which the need for nursing care services among the LTCI beneficiaries is equal, level 3 beneficiaries should use services provided by long-term care hospitals because they are not permitted to use the institutional care services in the Korean LTCI policy.

Secondly, the number of beds per 1,000 elderly persons increased to 15.3 beds in 2010, the greatest increase rate among all OECD countries [1]. The average annual increase rate of the number of beds in the long-term care hospitals was 20.8% from 2007 to 2010 [25]. This rapid rate of increase led to a 14.1% rise in the average annual rate of the LoS for Korean senior citizens over the same period [3], and it could be related to an increase in the LoS of LTCI users.

However, we can’t suggest that the introduction of the LTCI program does not have an effect on the LoS of LTCI users. We found that the LoS of level 1 and level 2 beneficiaries decreased by 8.35 and 2.84 days, respectively. This decrease among level 1 and level 2 beneficiaries may be because level 1 and level 2 beneficiaries could choose the institutional care services provided by the LTCI program under the guidelines of the Korean LTCI program, and because the need for inpatient services of level 1 and level 2 beneficiaries was reduced.

In addition, the difference in out-of-pocket costs between long-term care hospitals covered by the Korean NHI program and long-term care institutions covered by the Korean LTCI program may result in a decrease in the LoS of level 1 and level 2 beneficiaries. LTCI beneficiaries admitted to long-term care hospitals paid 840 thousand Korean won (KRW) per month in out-of-pocket costs, while those who used long-term care institutions paid 485 thousand Korean won (KRW) per month in out-of-pocket costs [26]. This difference in out-of-pocket costs could cause level 1 and level 2 beneficiaries to select institutional care services instead of hospital services. The decrease in the LoS is consistent with the results of previous literature in which there is a substitution effect between long-term care institutional services and hospital services [15].

## Conclusions

This is the first study to examine the effects of the Korean LTCI program on the utilization of health care services covered by the Korean NHI program for the elderly population. This study demonstrates that the LTCI program reduces the LoS for level 1 and level 2, even though it does not reduce the LoS of level 3. The reason why there is an effect on the LoS of level 1 and 2 beneficiaries is that they can choose the institutional care services provided by the LTCI, and out-of-pocket costs of institutions are lower than hospitals. However, the reason why there is no effect on the LoS of level 3 beneficiaries is that they are not permitted to use the institutional care services under the Korean LTCI policy. Therefore, we recommend a modification in the LTCI system that facilitates the use of long-term care institutional services by level 3 beneficiaries without conflicting Korea’s LTCI principle to promote home-based care services instead of the institutional care services.

## Abbreviations

NHI: National health insurance
LTCI: Long-term care insurance
LoS: Length of stay
DID: Difference-in-difference
NHIS: National health insurance service
RPRC: Research project review committee
IRB: Institutional review board
CPI: Consumer price index
KRW: Korean won
